# Community Services and Social Involvement in COVID-19 Governance: Evidence from China

**DOI:** 10.3390/ijerph192215279

**Published:** 2022-11-18

**Authors:** Jianwen Ding, Jia Xu, Thomas Weise, Huan Wang

**Affiliations:** 1 School of Public Administration, Hohai University, Nanjing 211100, China; 2 School of History, Anhui Normal University, Wuhu 241002, China; 3 Institute of Applied Optimization, School of Artificial Intelligence and Big Data, Hefei University, Hefei 230601, China; 4 Sino German Talent Exchange Center, Hefei University, Hefei 230601, China

**Keywords:** community service, COVID-19 governance, social involvement, smart community, China

## Abstract

This study explores how the services provided by different types of Chinese communities varied in their impact on the social involvement of residents during the COVID-19 pandemic. The literature revealed problems caused by travel restrictions, including using oversimplified measures for grassroots governance, which might result in decreased residents’ social involvement during COVID-19. We argue that the services provided by “smart communities” in China not only adhered to the COVID-19 pandemic governance, but also promoted the social involvement of residents. Using a case study approach of the smart community Fang Xing and the traditional community Qili Tang, both of which are located in China, this article compared the traditional and smart community services based on 122 interviews with residents and frontline community staff members. The findings suggest that while the traditional community decreased the residents’ social involvement by restricting certain services during the pandemic, the smart community was able to apply COVID-19 governance measures, considerably increasing the residents’ social involvement. It offered an attractive option for residents to act as community service managers, and it prepared them for local-level pandemic governance. This study provides an understanding of the relationship between the community services and the residents’ social involvement in terms of the community services. The smart community model can act as a reference for international community development during pandemic governance.

## 1. Introduction

The COVID-19 pandemic has caused population loss throughout the world. Many people also experienced social exclusion as a result of the virus infection and lockdowns [1,2,3]. However, we found that residents in a new kind of community—the smart community—experienced increased social involvement during the pandemic in China. Smart community services have made remarkable advances domestically, particularly with the use of new technologies and new kinds of services that were developed to control the spread of the virus and improve social involvement [4]. This kind of community provided the residents with various services to help them persevere during the pandemic, such as offering food and package delivery services to quarantined apartments [5,6]. Thus, communities have enhanced pandemic governance measures, while simultaneously offering distinct services that increase social involvement (i.e., the residents’ degree of community participation) [7].

The experience of local-level pandemic governance has proven that there were problems caused by measures such as travel restrictions and using oversimplified measures for grassroots governments [8,9]. However, it must also be recognized that such measurements decreased the residents’ social involvement during this COVID-19 crisis [10,11]. Therefore, we examine how two types of Chinese communities, traditional and intelligent, differed regarding the impact of their community services on the residents’ social involvement in the context of pandemic governance.

In recent years, China has introduced the “smart community”, a new type of advanced community that organizes, delivers, and monitors most of its services through online platforms [12]. Instead of relying on personal contact, such as in traditional communities, smart communities provide services, such as arranging home deliveries through the use of information technology [13,14]. Online platforms function as information processors that receive messages from the residents through their registered personal IDs and relay this information to the frontline community staff for further action to be taken [15]. They also function as information terminals that analyze the data collected from the residents, such as their travel and medical records, to identify the services that certain residents might need [13,15,16]. The online platforms also help to organize online medical services for the residents [15]. They can, for example, book online video consultations with doctors who will either prescribe medications that the patient can order online for home delivery or transfer patients to a clinic or hospital [15,17,18]. The platforms can also analyze the match between the residents’ requirements and the received services and use the residents’ feedback to improve the services [14,15]. The residents can access online platforms through their personal computers, mobile applications, quick response (QR) codes, and WeChat [19]. Although the platforms are developed and supported by software companies, they are managed by the communities themselves.

IBM’s “Smarter Planet” initiative, which was introduced in 2008, was the inspiration behind China’s smart community concept [20,21]. In 2014, China’s Ministry of Housing and Urban-Rural Development proposed guidelines for the development of smart communities [12]. These guidelines include the eligibility criteria for becoming a smart community and the assessment criteria, which state that any smart community resident should be able to receive proper, timely service at an appropriate location in the community [15,19,22]. Furthermore, China’s Software Evaluation Department created a smart community service evaluation system based on four dimensions: it offers smart services; it provides smart management and maintenance; it applies smart application platforms; it collects smart resources [19,23]. The smart community concept is becoming an increasingly popular community development trend in China, especially in regions with adequate budgets [19,22]. Cities such as Beijing, Shanghai, Ningbo, Shenyang, and Changsha have attempted to develop smart community pilot projects since 2013 with support from their municipalities [14,15,24]. At the time of us writing this, over 80% of the Chinese cities had at least one smart community [22]. Many municipalities are attempting to increase the support that the community receives by applying the smart community concept [25]. We argue that this novel community model may have greatly impacted the residents’ social involvement during the pandemic by helping to reduce their dependence on human resources, thereby decreasing the amount of direct personal contact and the infection risk.

This study provides a better understanding of the relationship between the community services and the residents’ social involvement in terms of the community services. The smart community model showed its advantages in local pandemic governance, and its original contribution to the area offers a form of necessary and continuous community services to the residents, and it aims to serve as a reference for the community service development models of other states, particularly in places where the public community services are underdeveloped. This study highlights the development that a community may go through as well as the impact of the smart services on the society, which are significant to providing community services for the residents and improving their quality of life and social involvement, especially during the pandemic governance period, while traditional community services cannot compensate for the unmet needs. This study contributes to the literature by proposing a connection between social service and more programmatically focused areas of community development such as smart community service and social involvement, which could be considered a good reflection for the use of the community change theory (Kelly, 1968). In addition, this study also offers a reference for future community development with better services while decreasing the infection risks in the event of a public health crisis.

This study measured the performance of community services, including smart and traditional communities, and we asked how this performance affected the residents’ social involvement to understand the relationship between pandemic governance and the community services provided during the pandemic at both the institutional and empirical levels. The comparative analysis is based on a case study of the smart community Fang Xing (Community F) and the traditional community Qili Tang (Community Q). Both of them are located in Hefei City in Anhui Province, China [25]. Anhui was one of the provinces that was least affected by COVID-19 in China, mainly due to its highly successful community-based COVID-19 governance measures, which is why we selected this province for the analysis. The target population of this study is the residents (including the volunteers) and the frontline community staff members in both Community F and Community Q. Therefore, this study conducted 122 semi-structured interviews with the residents and the frontline community staff members in both of the types of communities, in which they introduced the analytical framework in terms of the dimensions of the community services and their integration with the COVID-19 governance measures, which was followed by an analysis of the residents’ social involvement and the costs and savings of the two types of communities.

## 2. Literature Overview

### 2.1. Implications for Community Change Theory from the Literature

The literature on urban community dynamics is replete with examinations of the process of decline, which is evident in the early and contemporary examinations of community life [26,27,28]. With the ability of residents, community-based organizations, and policy makers, the examination of the intervention and factors that promote positive change in communities and community services can undoubtedly be found [29]. A growing body of literature has begun to document encouraging evidence about the interventions and factors that contribute to communities undergoing positive changes, including community development corporations, comprehensive community initiatives, and community residents [30,31,32]. To present the information that could be learned about for the community change interventions, the literature evaluates the different types of community development with regard to community change, which focuses on improving the community services within the specific strands [33]. The theory of community change underlies evidence of areas of intervention for community development, such as community building, housing, and economic development [34], while other research on the traditional analysis of community development focuses on the individual level, including social services and employment on the basis of the community change theory [35]. Many studies have also focused on the connection between social capital and more programmatically focused areas of community development, such as community care service and community building [36,37]. In addition, we also found that the research has proposed outcomes on the relationships between the social networks and social ties that contribute to serving a larger purpose, which indicates that neighbor behavior is the key element to influencing the development and maintenance of social cohesion [38,39]. This analysis presents good reflections on the current study based on the community development theories.

### 2.2. COVID-19 Governance by Local Chinese Communities

Public health and pandemic research has analyzed and discussed local communities’ COVID-19 governance. While most studies have focused on pandemic governance effectiveness [4,8], others have compared different community service dimensions [11,40,41]. The studies have highlighted the advantages of having local communities in COVID-19 governance by tracking the residents’ travel history and limiting their travel to COVID-19 seriously infected regions [8,9]. For instance, the Chinese COVID-19 online information platform includes Chinese “close circle” management records, which provide the medical records of the infected patients [42,43]. The platform also assists in supervising the infected individuals’ physical isolation, which can help decrease the spread of the virus [9,44]. However, other studies argue that the governance of traditional communities during the COVID-19 outbreak was limited because many community services were suspended without a long-term plan or comprehensive service management system [45,46,47]. For example, home care services were suspended due to the lack of human resources available for their provision [48,49].

### 2.3. COVID-19 Governance by Smart Communities

Smart communities utilize intelligent technology to efficiently provide services while maintaining a safe physical distance between the residents and the service providers [9,50]. For example, using the community’s online platform, adult children can order meal services for their elderly parents, make payments online, and offer feedback [51]. In addition, young children can receive free online classes, such as singing and dancing classes, which provide parents with some respite [9,52]. The smart community covers both the public service spaces and the residential areas and monitors the service provisioning process in real-time. The residents’ satisfaction levels are analyzed after the services have been provided [22,53]. Some studies have shown that a smart community can provide not only services that enhance daily living activities, but it also provide measures for pandemic control, such as digital body temperature detection [41,53].

In the comparisons of the pandemic governance of traditional and smart communities, the researchers have proposed that smart communities are more effective in governing during pandemics without restricting the service scope or downgrading its quality as they take advantage of advanced digital technologies [5,8,54,55]. Smart communities can also be considered to be a dynamic database for municipalities [56]. Since the COVID-19 pandemic began, they have collected and analyzed the health conditions, clinical diagnoses, treatment records, virus infection histories, and vaccination records of their residents to support the municipalities with verifiable evidence, facilitating the localization of potential virus-spreading risks for the governments [46,53,57].

### 2.4. Community Services’ Impact on Social Involvement during COVID-19 Outbreak

Social involvement in community services is defined as the participation in any part of a service-providing process that meets the residents’ requirements and contributes to service management in the community [58,59]. Previous research has classified community social involvement into different models, such as the voluntary and non-voluntary models and the institutional and practical models, arguing that community social involvement is a powerful impetus for community development [4,60,61].

Over the last two decades, the researchers have debated the assessment of the conditions for social involvement for offering community services [62,63]. Some studies have proposed specific indicators that should be included in the assessment procedures [64], such as the efficiency, frequency, and consequences of social involvement, including changes in the residents’ unmet needs [65]. However, other studies have argued that specific indicators are inadequate to assess the social involvement conditions, and that the actual outcomes must be examined through public crises [62,66]. Compared to traditional communities, smart communities can increase the residents’ social involvement via digital intelligence technology [55,66,67]. Chinese smart communities establish their information platforms by involving the residents online as a primary step, through which they collect user-generated information, create the personal identities in the system for each resident, connect to the residents’ social network accounts, and analyze their preference for social contact in the community [68,69]. The existing literature defines a smart community as a “dual system” that encourages the residents to not only post their service requirements but also offer services that have been requested, thereby allowing residents to involve themselves in the community service process and increase their contact with service providers in multiple ways [70,71,72]. Therefore, the digital platform identifies each resident’s living conditions, their general needs for daily living, and the family relationship in the system, and it offers targeted services to different people [67]. This may help the residents develop a better sense of participation in and identification with the community. These smart communities are expected to improve their social involvement conditions and their residents’ quality of life while also promoting local-level community development [68].

### 2.5. Research Gap

Previous studies have focused on the COVID-19 governance measurement of either the traditional community or the smart community, but they lack comparative outcomes between the different types of communities, either in China or through conducting global comparative research for community development in different countries. Additionally, the literature has focused on the impact of the technological applications of smart community services [55,73,74]. However, a systematic analysis of the relationship between the community services and social involvement during health emergencies is lacking.

## 3. Research Design and Data

Our study analyzes the distinct impacts of the traditional and smart communities’ COVID-19 governance with regard to the community services on their residents’ social involvement. We argue that pandemic governance necessitates the implementation of physical isolation measures without socially excluding people during serious COVID-19 outbreaks. Although Chinese communities made significant contribution to controlling the spread of the virus, thus allowing residents to return to normalcy more quickly than they have done in most of the other countries [75,76], their residents still suffered due to the restricted social involvement. We propose that traditional and smart communities differ in terms of their pandemic governance, and thus, the social involvement of their residents also differs through there being a greater reliance on either human resources or technology, respectively, in offering the community services [74].

Community F (FangXing Community), which has been a pilot smart community since 2015, covers an area of 11.8 km^2^, with 11 residential regions and 36,000 apartments. At the time that the study was conducted, it had a population of seventy-four thousand residents, including three hundred and forty individuals over eighty years old, two hundred and ninety-five social assistance benefit (DiBao) recipients, three hundred and forty-two individuals with disabilities, and nine individuals with mental or psychological disorders. The provincial-level local government office is located inside the community [77]. Community services are provided by approximately 30 formally employed staff members and several volunteers and part-time workers. In 2020, Community F had one suspected COVID-19 case. Over 300 residents living in the same building as the identified case were required to self-isolate in their apartments. No other residents in the surrounding buildings were infected, and the restrictive measures prevented the virus from spreading in the community.

Community Q (Qili Tang Community), which is a traditional community, covers an area of 21 km^2^ with 40 residential regions. At the time the study was conducted, it had a population of 210,000 residents with 1009 individuals over 80 years old, 201 social assistance benefit recipients, and approximately 800 individuals with disabilities. Approximately 50 staff members had formal contracts to work in the community. In 2020, Community Q had one COVID-19 case, and approximately 350 residents who lived in the same building were required to self-isolate in their apartments. No other residents in the surrounding buildings were infected.

We propose that smart community services can, in principle, promote both isolation and social involvement since this novel type of community can provide different categories of autonomous services. Through utilizing their technology and online information platforms, smart communities can help to reconceptualize governance measures during health emergencies and maintain social involvement. Therefore, we analyzed three dimensions that affected how the community services were provided during the COVID-19 outbreak: (1) COVID-19-related governance measures, (2) social involvement-oriented community services, and (3) budget costs. For the first dimension, we differentiated between four indicators that were implemented during the pandemic from 2019 to 2020: distance supervision, health condition governance and residents’ isolation, public opinion guidance, and restrictive measures on basic daily living activities. For the second dimension, we analyzed the services that supported the pandemic isolation and care services in the community. For the last dimension, we explored the community costs and budgets for COVID-19 governance equipment and human resources and their savings from utilizing either human resources or technology in the process.

### 3.1. Effective Pandemic Governance Measures and Adequate Social Involvement Services: Technology Use in Service Provision

To overcome the negative effects of the lockdowns, local communities attempted to offer residents the necessary social involvement services, such as home care services. However, empirical research has shown that despite the extensive spread of COVID-19, some residents may still have been unwilling to follow strict lockdown measures because they preferred to have “normal” community services [41]. Stricter COVID-19 governance measures combined with limited social involvement might even prompt residents to seek social contact, particularly when the measures are implemented for a long period.

Therefore, communities that have implemented effective pandemic governance measures while also providing adequate social involvement services for residents may discourage the residents’ incompliant attitudes toward COVID-19 governance measures. The problems associated with pandemic governance measures could in part be mitigated if the residents’ normal daily living needs are met and if they receive adequate community services to compensate for the lockdown-related inconveniences. If the services are primarily provided through digital technologies with human personnel providing auxiliary support in some services, the residents may have a greater incentive to embrace these services since this strategy both lowers the infection risk and meets their daily living and social needs. Thus, we assume that smart communities, such as Community F, provide effective pandemic control and promote adequate social involvement.

### 3.2. Effective Pandemic Governance Measures but Fewer Social Involvement Services: Human Resource Use in Service Provision

Pandemic governance and social involvement may be at odds; for instance, a community may close the social interaction spaces during a lockdown. Communities might become more conservative when they are offering community services because of the lack of effective measures or technologies to provide such services, as they rely heavily on human staff. However, when infected cases appear in a community (such as in Community Q), the traditional human resources may be insufficient. More people are needed to perform even basic living services such as food delivery and garbage disposal. Such a community might disregard the residents’ need to maintain social contact, which could lead the residents to meet their social needs in other ways, and thus, increase the virus infection risks. Therefore, we assume that communities such as Community Q may provide effective pandemic control but insufficient socialization opportunities.

We conducted a comparative empirical study to analyze the relationship between the COVID-19 governance and the social involvement of the residents in both of the community types. We conducted 122 semi-structured in-depth interviews. The sample size of this study depended on our research focus with regard to information saturation. We decided that our sample size criterium along with how much information was gathered from the interviews was fulfilled [78,79]. The information that we intended to gather depended on our research question for this study [80]. The interviews were constantly in progress to achieve information saturation, and we found that information saturation was reached after we had conducted 102 interviews in total based on our interview outlines, and no more new information was added, which is a valuable fact to include in this study [79,80]. Then, we conducted an extra 20 interviews in order to double-check the information that we had gathered [81].

Our interviews consisted of 42 in-person interviews, including 31 residents (10 of them were also volunteers) and 11 staff members; 30 telephone interviews, including 20 residents (10 of them were also volunteers) and 10 staff members; 50 online video interviews, including 40 residents (10 of them were also volunteers) and 10 staff members from June to August 2020 in both of the communities. It should be noted that both of the communities had experienced the travel restrictions by the time we conducted the interview, which means the current study focuses on the period in which the travel restrictions were lifted instead of the first few weeks of the pandemic. Thus, our main interview method was in-person, the online video type was our second choice, while the telephone interview is listed as the last choice, which is also a compensatory interview choice in order to confirm the information that we obtained by the first two methods in the interviewees [82,83]. In order to avoid a different sense of tension that may have been created by different interview methods, we made sure to make an appointment before each of the online and telephone interviews, and we made sure that these interviews could be conducted in a comfortable and safe environment for the interviewees [84,85]. The age of the interviewees ranged from 18 to 85 years, and 55% of them were female. The resident interviewees included the care-dependent elderly residents, the mothers of young children, individuals with disabilities, social assistance benefit recipients, volunteers, and ordinary residents. We present also the general sociodemographic information for the interviewees in Table 1 and Table 2.

Each interview lasted for at least 30 min. The in-person interviews were held in meeting rooms, and the audio was recorded for later transcription. Consent was obtained from all of the interviewees prior to the recording. Similarly, the online interviews were conducted and recorded via online platforms. The phone interviews were also audio recorded. Consent was obtained from all of the interviewees prior to the recording, and an interview manual was used for the interviewing process. This study recruited participants for the interviews by sending invitations and application forms to both of the communities and publicly recruiting participants when they were willing to take part. We then checked all of the applications and selected participants based on our criteria for this study. The selected participants were from 18 to 85 years old and could cognitively communicate with the interviewers. The resident participants should have lived in the community for more than 6 months, with them having lived at least two-thirds of their time in their communities in the three months prior to the time we received their application. The staff members who participated should have least worked in the community for 6 months, while their work had to be related to community services all of the time. In addition, we also established a priority criterion for the participant selection [82,86,87]. We regarded that residents who had lived for longer and/or received community services were prioritized over the other potential participants, while the frontline community staff who had worked for longer and/or the supervisors among the frontline community staff were prioritized [83,88]. We sent a final decision letter to potential participants. However, all of the participants were able to withdraw at any time for any reason. Ethical approval was obtained, and this study was conducted in accordance with the ethical principles regarding human experimentation in the Declaration of Helsinki. The semi-structured interviews used a prepared topic guide as a starting point, which included prompts and open-ended questions covering mainly two topics: the effectiveness of the COVID-19 governance measures and the degree of the residents’ social involvement in the communities or community services. These questions were also posed to the service personnel and managers, along with questions regarding the cost and budget of the pandemic governance. Socio-demographic information about each interviewee was gathered in a short questionnaire before each interview. The data analysis was based on the framework method [89,90]. The transcripts were initially segmented by two authors (J.D., J.X.) of this paper, from which JWD selected codes for the analysis of the research question. J.X. analyzed the data and discussed the analysis in the regular online meetings. The degree of the residents’ social involvement in the communities or community services was further discussed with T.W. and H.W. The transcriptions were analysed using the data analysis software Nvivo (Nvivo 12, Burlington, VT, USA) and Microsoft Office (MSO: 16.0.4266.1001, Redmond, WA, USA). This study was part of a larger research project involving community development and poverty risk in China, and we received ethical approval from the ethics committee of the Anhui Normal University. Written informed consent was gathered from each interviewee. Apart from the interview data, the basis of our empirical analysis included institutional regulations from the Chinese Center for Disease Control and Prevention, the Chinese National Health Commission, the Chinese National Healthcare Security Administration, and the Chinese COVID-19 online information platforms.

## 4. Results

### 4.1. Comparative Impact of Pandemic Governance and Social Involvement: COVID-19 Governance Measures in the Community

#### 4.1.1. Health Condition Governance and Residents’ Isolation

Smart community F: This community took measures to supervise people’s health conditions and isolate the infected patients according to three steps. Firstly, digital technologies such as online platforms and chat groups provided the residents with updated virus infection information, including the exact location of the local isolated apartments where residents had to stay at home and observe any changes in their health conditions. Secondly, the community asked all of the residents to record their travel history on the online platform so that it could supervise and analyze the infection risks for all of the residents. We argue this action was effective since most of the infection cases resulted from traveling. When the travel history can be supervised in a timely manner, without face-to-face contact, the communities may achieve positive pandemic governance outcomes, as mentioned by one interviewee.

*“I traveled through a place with relatively high risk of virus infection. I remember I was advised to stay at home for 14 days of home health condition supervision immediately after I returned home. I needed to upload updated information on my body temperature and the nucleic acid amplification test (NAAT) results to the online platform in the following days. But it was amazing that I got responses on the platform to my updates as someone online replied to my questions quickly.”*

(FX11, male, resident, 30 August)

Thirdly, Community F tracked the residents’ health status through pharmacies, and unlike the traditional communities, they conducted this supervision online. When the residents purchased medicine online or in person, they completed an online form to report their personal information and purchase the details. These measures have been shown to effectively help supervise the changes in the residents’ health conditions without increasing the infection risk to staff.

Traditional Community Q: We found that over half of the residents did not know the location of the isolated apartment. Due to limited human resources, the personnel could only inform and supervise those who had to be isolated. Furthermore, the service personnel updated the travel history of Community Q residents through door-to-door visits and WeChat. However, some residents were not at home, and others could not be reached via the phone. The residents were responsible for reporting their travel history, but we found that not all of them followed this guideline in practice. One staff member expressed her experience as follows:

*“It’s very hard to supervise residents’ movements in the community. We have to knock door-to-door or call them one by one, and we advise them to not meet with other people unless it’s necessary. But some act against our advice, despite saying ‘yes’ to us. We don’t have that many colleagues to do this job. We’ve already been working over 10 h a day during the first wave of the pandemic, it was really exhausting.”*

(QLT50, male, staff member, 4 July)

Additionally, compared to Community F, the Community Q residents still obtained medicine from stores, and even antibiotics through transactions on paper. Thus, it was difficult for the community to obtain an overall picture of the residents’ medicine purchases.

#### 4.1.2. Public Opinion Guidance

Both Communities F and Q performed well in guiding the public opinion by organizing chat groups in applications such as WeChat and QQ to deliver messages on how the residents could support the pandemic control measures. These online chat groups covered approximately 99% of the residents, or at least one member in all of the families in both of the communities. However, we noted there were 300–500 people in each chat group, and generally, only one group manager, and 3–5 staff members from the community who could answer questions. Many residents commented simultaneously in the chat window; thus, most of the questions were not answered in a timely manner. Many of them were left unnoticed or were covered by new questions, despite the efforts of staff who worked overtime, sometimes until midnight and through the weekends. The Community Q residents often complained during our interviews that their questions were left unanswered and that their problems remained unresolved since these chat groups were the main way to express their needs (QLT27, female, resident, 25 August; QLT20, female, resident, 20 August).

Other than the chat groups, Community Q focused more on traditional measures to guide public opinion, such as using broadcast vehicles to deliver urgent information and posting lockdown notices at building entrances. A community manager said:

*“We realize it’s really hard to guide public opinion by only talking to residents during the pandemic. My colleagues spent hours per day providing information on pandemic governance measures, which is something hard to understand for residents, and we have no idea how the residents think and what they need.”*

(QLT63, female, community manager/staff member, 5 July)

Smart community F: We found that Community F performed better in guiding the public opinion by uploading the residents’ questions and problems from chat groups to their online platform. Firstly, the platform classified the messages into different types, marked them as tasks, and then sent them to the work calendars of the relevant staff. Subsequently, the staff managed these tasks, while the reminders for the pending tasks were continuously sent. Consequently, the residents’ messages were not easily lost, the messages and questions were responded to quickly, and the residents’ problems were resolved faster when they were compared to those in Community Q. The residents could also use their individual IDs to log into the platform and post questions (through text or voice messages) to the staff directly, which were then transferred to the work calendars. Moreover, to present updated information on pandemic governance, Community F installed equipment across the community, such as 15 electronic screens and 100 public information boards. These intelligent measures positively impacted the guidance of public opinion, particularly during the first wave, as residents were closely connected online.

#### 4.1.3. Restrictive Measures on Basic Daily Living Activities

Restrictive measures on daily living activities are essential for pandemic governance, especially when a community has verified or suspected COVID-19 cases. Here, we discuss the extent to which the residents followed these restrictions, leading to changes in their normal living patterns and unmet daily living needs, and how well community services met their residents’ needs.

Smart community F: The smart communities encouraged the residents to follow restrictive measures to promote a balance between the residents’ daily living needs and the necessity of pandemic governance [40]. We found that the messages about the basic daily living needs, including food shopping, package delivery, and garbage disposal, among others, received a prompt response. The system avoided missing the messages by creating system backups of feedback during the isolation period.

As a result, a few residents complained about unmet basic daily living needs. Meanwhile, the volunteers who offered the services to support restrictive measures could reduce their working hours by 20% when they were compared to those in Community Q (FX13, male, resident/volunteer, 13 August). The Community F residents understood the restrictive measures and developed a sense of intuitive cooperation. We also found that while the residents had complaints and bad service experiences, they could present them as feedback on the system. Most of these complaints were resolved quickly with the help of the platform. Thus, residents were more likely to be integrated into the community’s management. One resident expressed this as follows:

*“My family felt frustrated when we were notified that we couldn’t leave the building for two weeks. It seemed like we needed everything suddenly, and we were locked there. Even my cat needed food. We called the community center, but no one could be reached. I guess there were just too many people calling them. Luckily, we contacted staff online, and it was amazing how quickly they replied. I could upload what I needed to the system, and then I received my items at the building entrance. Although I felt sad when I saw the security guards protecting the entrance wearing horrible white protective clothing. It felt like we were all sick. But I felt better when community staff reached out to me via a video call from the online system to comfort me and give me some peace.”*

(FX09, male, resident, 28 August)

According to the records of the restrictive measures implemented in the isolated building, two residents from different apartments had fevers during the isolation period in Community F. These residents received online medical services from the platform, and medicine was delivered to their apartments. Medical staff administered the NAATs to these residents every two days for two weeks, instead of one NAAT every three days as was administered to the other residents (FX14, male, resident, 28 August). None of the family members living in the same apartment as the suspected infected individuals were permitted to leave the apartment or go to the main entrance. However, a delivery of daily living support packages were delivered to their apartment door. We investigated whether the other residents in the isolated building had a cough or cold during this period, however, the results show this was not the case. A frontline community staff member believed that wearing face masks for in-person interactions was the main reason for preventing spread of the virus (FX54, female, staff member, 12 July).

Traditional Community Q: Some of the Community Q residents also experienced restrictive measures due to a COVID-19 infection case. Over 300 people in the building where the case was identified were isolated for two weeks. Similar to Community F, basic living services, such as vegetable purchases, were provided by staff and volunteers. The residents contacted the service personnel daily and communicated their needs via telephone and WeChat messages. The volunteers recalled they went shopping more than 10 times per day, delivered goods to the residents, and called the residents to pick up the deliveries at the building entrance. Sometimes the residents’ needs could not be met due to misunderstandings or a lack of timely contact with the staff/volunteers (QLT32, female, resident/volunteer, 20 August). Over 40 disputes occurred with the isolated residents during this period, which the community director and her team had to resolve in person. Consequently, human resources played a key role in the pandemic governance measures in Community Q.

Similar to Community F, four residents from different apartments in the isolated building had fevers and headaches during the isolation period. They also received NAATs every two days from the medical staff. However, they did not have access to online medical services, and medical staff needed to check their condition every two days in person instead of online (QLT63, female, community manager/staff member, 5 July). The family members living in the same apartment were not allowed to leave the apartment, and their garbage was removed by the staff. There were also no additional cases of coughs or viral colds during this period.

We conclude that both of the communities relied on human resources in this period, however, Community F could better organize the services due to its online platform. Although both of the communities had a similar percentage of volunteers and staff, those in Community Q had 20% longer working hours. Over 30% of its volunteers and staff complained about feeling exhausted in this period (QLT63, female, community manager/staff member, 5 July; QLT23, female, resident/volunteer, 20 August).

### 4.2. Social Involvement-Oriented Community Services

#### 4.2.1. Services Supporting Distance Supervision

Smart community F: Community F utilized its digital services as preventive measures to decrease the spread of the virus. For example, the online information platform controlled the entry/exit of people by identifying vehicles and the people that passed through the entrance and analyzing whether they complied with isolation guidelines. Infrared body temperature measurement equipment which was placed in different areas of the community could trigger alerts as soon as high body temperatures were detected. The frontline community staff then responded to handle this risk. This increased the social involvement of both the residents and staff, as expressed by a manager:

*“I’m very confident in this online platform because our staff can precisely locate residents’ movements in public places with the help of the platform. We were online for 24 h, taking turns in 8 h shifts during the pandemic. Additionally, the platform supervises sanitation information of buildings. For example, it tells me whether the supermarket is too crowded.”*

(FX11, male, resident, 30 August)

The Community F residents were required to register their health conditions, travel records, and vaccination records using QR codes which had been positioned in public places. While this was similar to what was performed in most communities, Community F did not need the staff to individually check the QR code results in person. Its online platform received the QR code results as soon as people scanned the codes. When the results indicated a virus infection risk, the platform alerted the staff. Thus, Community F was able to reduce its on-the-ground staff by approximately 80% and implement more online services.

Traditional Community Q: Here, many volunteers assumed temporary duties related to the pandemic governance services, such as body temperature checks at the entrances of most of the public places. Community Q also placed QR codes at almost all of the places that required people to reveal their health status. However, staff—either wearing face masks or protective clothes—checked the results and residents one by one. Therefore, Community Q had more staff in the field. Instead of supervising the residents’ activities, it also closed some public spaces that had been used for parties or dancing by older residents or as playgrounds for children as well as stores that sold flowers, snacks, and accessories to restrict the residents’ opportunities to gather. Although these measures decreased the risk of spreading the virus, they also increased the amount of social exclusion by restricting social contact.

#### 4.2.2. Community Care Services

It is important to explore the extent of the care services received by care-dependent older residents and the degree to which their care needs were met while the COVID-19 pandemic governance measures were in place. In principle, these people depend on either community care services provided by care homes, home service deliveries, or family care work, which is mostly performed by female family members [48]. However, both of the communities closed their care homes and decreased the number of home care delivery services owing to the pandemic governance requirements. Moreover, family caregiving was inaccessible to older people without family members or whose family members lived far away [7].

Smart Community F: Community F identified 79 care-dependent older adults who did not have family care services and offered them an “intelligent bracelet”. This bracelet was connected to the online information platform, and it transferred information about the older residents’ location and movement. Thereafter, the staff could contact them when it was necessary. For instance, a 76-year-old resident who was living alone showed a sudden increase in his daily movements, however, according to information from the platform, he had difficulties with physical movement (FX22, male, resident, 13 August). The frontline community staff visited him immediately and found that he had been so bored that he had forced himself to go outside, and he became lost. After this incident, the frontline community staff sent him videos and news broadcast channels through the information platform and called him regularly to check on his condition.

The intelligent bracelet could also send emergency calls to the platform on behalf of the older residents, after which the services could be offered to them as needed. The home services and delivery-specific care services could also be provided through the bracelet when they were necessary. As expressed by one staff member:

*“Thanks to our intelligent bracelet and information platform, we noticed some poor and care-dependent older people stayed at home without consuming electricity for days or without using water, which meant they might be facing problems in their daily living and/or have deteriorating health conditions. We offered them help and services soon after we received notice through the platform. Sometimes we would deliver bread, rice, oil, vegetables, and medicine for free to their homes. These people cannot ask for help since some of them have serious physical disabilities and others have mental health problems, so we have to determine their needs.”*

(FX56, female, staff member, 16 August)

Therefore, the care services in Community F contributed to the maintenance of the social involvement of care-dependent older people and helped to fulfill their care needs, autonomously. Although the isolation measures restricted these people to their homes, the smart digital technology connected them to the social networks [74].

Another crucial issue that was revealed in our interviews was the boredom and loneliness that was experienced by older residents with disabilities. Their concerns were reported through the information platform. The staff offered them detailed information on the nearest real-world spaces, online chat rooms, and timelines where they could interact with other people (FX56, male, staff member, 17 July). Furthermore, Community F offered the residents free online courses for mental health development. Doctors specializing in psychological disorders helped the residents to resolve their negative feelings and reduce their fear of COVID-19. Other courses were offered to students of different ages and teachers. Therefore, we believe Community F greatly improved the residents’ social involvement by offering daily living services during the pandemic.

Traditional Community Q: The care-dependent older people were sent to live with their families because the care resources in the care homes were limited and the home service delivery was suspended. In other words, the delivery of the emergency care services by the community was not guaranteed, effectively making family members responsible for the delivery of the services for the care-dependent older adults. We argue, therefore, that during the pandemic, the care services in traditional communities not only decreased the care recipients’ social inclusion but also increased the family members’ care burden [48]. However, the family members could not always provide the timely delivery of care services, as in the case of family members who did not live with the care-dependent elderly individuals. As expressed by an adult daughter:

*“I should have delivered lunch and dinner every day to my mother, who lives in a neighboring sector in our community. Normally, it takes me five minutes to do so, but I couldn’t reach her any more due to the restrictive pandemic control measures. [The community care homes] stopped food delivery services, as well. I couldn’t leave her there alone unless I moved to her apartment, but I also have a young boy to take care of. We got no help, and we had to handle this problem by ourselves, which I’m still working on.”*

(QLT27, female, resident, 25 August)

In summary, our findings indicate that Community Q contributed weakly to social involvement in terms of the two measured indicators. In particular, its care services were almost completely dependent on care provided by the family regardless of the availability of the care provided by the family. Conversely, Community F effectively promoted social involvement through its intelligent services, and the smart platform substantially contributed to providing services to the residents. Furthermore, Community F invested time and money to establish the technological platform and change the community management patterns; it took approximately three years to create the online platform at a cost of nearly RMB 2 million (about USD 315,000). However, the community saved money that would have been spent on human resources investment. In contrast, Community Q did not spend its budget on technology investment, but instead on traditional resources, such as recruiting and training staff, and it took full advantage of the volunteers’ contributions to its pandemic governance. In the next section, we explore the differences between the two communities in terms of the pandemic governance costs and savings.

### 4.3. Community Costs, Savings, and Budgets for COVID-19 Governance

One of the most important intelligent infrastructure costs for Community F was the online platform. The software to support the platform cost around RMB 950,000 (about USD 150,000), and they received financial support from the Civil Affairs Bureau of Hefei. The software allows the online platform to analyze the residents’ information, such as by tracing their travel records and analyzing their health conditions, and it connects the platform with the central institutions’ data systems. The Civil Affairs Bureau of Anhui province allocated RMB 900,000 (about USD 140,000) to Community F to improve the platform. However, the community was responsible for organizing the resident services, receiving information regarding the residents’ needs and feedback, and improving the service quality. The costs of the other equipment, such as the video equipment, automatic temperature measuring equipment, automatic charging equipment, and intelligent bracelets, was approximately RMB 200,000 (about USD 31,500). Furthermore, the community’s yearly maintenance fee for the intelligent software is around RMB 60,000 (about USD 9450). Additionally, the municipality provides the community with RMB 50,000 (about USD 7900) per year to hire temporary staff (including the volunteers’ daily support fee) to conduct community services, especially during the pandemic.

One of the main reasons that Community F had a sufficient budget to develop its intelligent equipment and online platform is that the local government identified it as a model smart community, which could be a good reference for the development of other communities. Additionally, the local government intends to further establish more smart communities in the region. Therefore, both the city- and provincial-level governments included its intelligent infrastructure development costs in the central institution’s budget. As a result, Community F had more governmental financial support than the other communities did. In addition, Community F was able to match its residents’ needs by providing services through the online platform. This allowed it to offer better services and collect better feedback, which are the most important indicators for obtaining continued governmental financial support. It should be noted that both of the communities offer resident services for free, in particular, no extra fees were charged during the pandemic.

Conversely, as it did not have a central platform where the residents could communicate their needs and provide relevant feedback, Community Q allocated most of its budget to human resources. It established 67% more community management offices to handle the resident services and it had 37% more staff than Community F. The average annual per capita income level in Hefei is approximately RMB 95,000 (about USD 14,960) [77]. This implies that if Community Q eliminated 10 staff positions for two years, it could theoretically afford the online platform, and the system would continue to save on personnel costs. The municipality and other government bodies regularly provide basic financial support to Community Q as well as to the other communities in the city.

Community Q accumulates savings by taking advantage of the support from its many volunteers—79% more volunteers than the number of those supporting Community F. From our analyses, we argue that Community F lowers its costs because it needs fewer temporary personnel and volunteers, but its intelligent equipment and equipment maintenance fees are expensive. This investment can be an obstacle for other communities. The residents of Community F residents receive better support, whereas those in Community Q have to rely on patterns of traditional services, such as manual temperature measurements. Our interviews revealed that many residents would prefer to pay for some kind of intelligent service system that is similar to what Community F offers. The residents also expressed their confusion as to why Community Q does not apply the online intelligent platform (QLT32, female, resident/volunteer, 20 August; QLT30, female, resident, 2 July; QLT35, female, resident, 5 July). However, building a comprehensive intelligent system is expensive and time-consuming. It is also difficult for the governments to support such projects in regions with limited financial budgets. Nevertheless, this type of system could be a community development trend in the future, especially in regions at an adequate socioeconomic development level. It should also be pointed out that once the intelligent system software has been developed and tested in a sufficient number of communities, the per-community development cost could likely be significantly reduced. This makes pilot projects such as Community F very valuable for provinces and municipalities.

## 5. Discussion

### 5.1. The Inclusive Way of Development for the Smart Community

It has been argued that smart governance and services in the community are effective for community governance and service delivery to the residents based on the digitally online service platforms, as we have presented in this study. However, as the services are increasingly provided, digitally, what those people who are less technologically inclined should do in this era of smart technology is a significant question to think about. These people might suffer from exacerbated social exclusion due to their lack of access to online services [15] (Liu et al., 2021). Therefore, we argue that communities could be more inclusive in the way that they deliver their services by, firstly, keeping the traditional service delivery method as a window to people who are less able to access digital services. For example, the communities could keep in-person services in the community centre to answer questions from the residents on service delivery and organize the proper services that they need. Actually, this is not limited to the community, but it also refers to smart governance. For example, in the hospitals where the smart patients’ services have been widely practiced, they have kept traditional service ways for the people who have difficulties accessing to digital ones. Secondly, the communities could include volunteers to supply the residents with the digital services and help the residents to better understand smart governance in the community while also gathering information on the residents’ needs at the same time based on the volunteer organizations and other social organizations. Thirdly, the communities could deliver their digital services to the family members of people who have less access to smart governance in order to include more people in this new way of conducting community development.

### 5.2. The Effective Way for Traditional Communities to Develop Services

We found that most of the people who had faced difficulties in accessing public services were the disadvantaged populations. They are very unlikely to be living in a smart community. They may be afraid of social involvement because they are afraid of being discriminated against [15]. Therefore, whether the traditional communities act in their role in a more effective way in terms of service delivery is the next question of concern. We argue that digital services were indeed expensive in the early stages of their development, such as using the online platform for organizing the service delivery. However, it shows that these kinds of digital service measurements could last for many years, and they cause different levels of costs since the community can choose to equip them at different smart levels. Including the lowest level of digital service equipment does not mean that the community includes fewer smart services, it only turns out fewer service items instead. Moreover, we argue that digital service equipment could broaden the service coverage instead of limiting the smart services to one community. Which means that different traditional communities can be included in the service coverage of a smart community in the same city by sharing smart governance costs to some degree, either financially or by offering other kinds of support to the smart communities. Consequently, this could increase social equity in the field of community development in one region, which might result in the involvement of residents, particularly disadvantaged populations, who are very unlikely to live in a smart community. Therefore, it turns out that the ease of governance in smart communities does not cause the further social exclusion and marginalization of the disadvantaged population. Of course, a period of time needs to pass before the smart community broadens its coverage in the region. Nevertheless, the general trend of its characteristics with its development has presented its inclusiveness in China. In addition, we argue that local governments can also increase the traditional communities’ chances to increase their effectiveness of service delivery by sharing the community governance data with them and extending the coverage of the smart community services by offering financial support to the traditional communities. In addition, traditional communities may also take advantage of the service center by positively exploring more service items that meet the residents’ needs, such as homecare service delivery to older people.

### 5.3. The Generalization of the Smart Community

The COVID-19 pandemic has led to serious effects on the pandemic governance measurement changes in most of the Chinese cities, however, the levels of these kind of impacts are not similar. In the communities that have well-developed original infrastructure, such as smart community governance systems, it turns out that their pandemic governance outcomes are better than those in other communities. However, how far this kind of smart community in the region can be generalized depends mainly on the government support and the financial support from multiple levels of government. We found that Chinese communities are experiencing a reform with regard to developing communities with high amounts of technology. When certain communities make significant progress in their services, the relevant local government may have a good chance of receiving financial support from higher level governmental institutes (e.g., government at the provincial level or the central government) [91,92]. Therefore, local governments have tried to develop pilot projects for establishing smart communities if they are somewhat able to financially support the community [77]. In addition, there being support from social organizations, NGOs, and other charitable organizations in certain regions is also important for the generalization of smart communities. We found that traditional communities have tried to include the smart online services system step by step, and they have demonstrated good progress [93]. However, presently, smart communities have only been developed as pilot projects in some parts of China. The high cost of digital infrastructure could pose a challenge to building such communities, especially for local governments and communities in socioeconomically underdeveloped regions.

## 6. Conclusions

This study explores the differences between the traditional and smart communities in China in terms of pandemic governance and the social involvement of the residents during the COVID-19 pandemic, and it presents a reference for community development for other countries with regard to pandemic governance. It has been presumed that Chinese communities support COVID-19 governance either by focusing on preventive and governance measures or by decreasing their residents’ social involvement.

Our findings indicate that the communities can both apply the pandemic governance measures and promote their residents’ socialization. We found that traditional communities decreased their residents’ social involvement because they often restricted their services during the pandemic. In these communities, the family members were obliged to take on both the daily living services and family care tasks under the pandemic restrictions. Compared to the smart communities, the traditional communities are often slow to respond when they are providing the required community services.

In terms of the implications, our study suggests that the community services in smart communities promote their residents’ social involvement when the pandemic restrictions are in place. This community type depends highly on technology platforms and digital equipment to include the residents in the service provisioning process. It, thus, also offers an attractive option for the residents to act as community service managers. Moreover, smart communities prepare the residents for local-level pandemic governance and resolve the service problems more quickly than traditional communities do.

This study provides new insights regarding the local communities’ contributions to COVID-19 governance. It clarifies the common understanding of the relationship between pandemic governance and the social involvement of the residents. It also provides evidence for enhancing the community services and social involvement. Moreover, it suggests that it may be interesting for other countries to explore the intelligent technology used for effective COVID-19 governance since it can decrease the high dependence on in-person human resources. This study sets an example for future community development in order to better serve residents, while decreasing the infection risks in the event of a global public health crisis. However, it has its own limitations: (1) It was based on a qualitative method approach, and so it lacks evidence to support the casual relationship between the social involvement condition of the residents and whether the community belongs to smart communities. It also lacks quantitative data analysis results to prove this causal relationship with a large sample size. (2) There were time constraints for conducting the interviews. More interviews should have been conducted both after the travel restrictions were lifted and instated due to the COVID-19 pandemic governance in these targeted communities.

Future research should firstly analyze the pandemic governance and the social involvement in more Chinese communities. In particular, it would be valuable to focus on communities that are in the process of becoming smart communities. We would also like to analyze the historical changes in community pandemic governance to identify the patterns in Chinese history. Secondly, we would like to explain the development of smart communities and their services for the residents by using quantitative research methods with data from surveys, which focuses on analyzing smart community generalization in different regions of China. Moreover, we are interested in performing a longitudinal data analysis on how much the smart community services impacted on the residents’ life changes during the pandemic governance in China and other countries.

## Figures and Tables

**Table 1:** Sociodemographic information for resident participants.

Variable	Category	Number	Proportion %
Gender	Male	42	46.2
	Female	49	53.8
Age	18–35	21	23.1
	35–60	18	19.8
	60–65	19	20.8
	65–70	15	16.5
	70–80	11	12.1
	>80	7	7.7
Family Member	1	16	17.6
	2	23	25.3
	3	26	28.6
	4	17	18.6
	>4	9	9.9
Employment Status	Employed	43	47.2
	Retired	48	52.8
How long they have lived in the community	6 months to 1 year	12	13.2
	More than 1 year	79	86.8
Whether they live alone	Yes	16	17.6
	No	75	82.4
House Ownership	Self-own	78	85.7
	Rent	13	14.3
Whether they are volunteers	Yes	30	32.9
	No	61	67.1
Which community they live in	F	44	48.3
	Q	47	51.7

**Table 2:** Sociodemographic information for staff member participants.

Variable	Category	Number	Proportion %
Gender	Male	13	41.9
	Female	18	58.1
Age	22–35	21	67.7
	>35	10	32.3
How they have been employed	Less than 1 year	2	6.4
	1–3 year/s	13	41.9
	More than 3 years	16	51.7
How long they have worked in the community	Less than 1 year	4	12.9
	1–3 year/s	16	51.6
	More than 3 years	11	35.5
Whether supervisor among staffs members	Yes	11	35.5
	No	20	64.5
Which community they work at	F	14	45.2
	Q	17	54.8

## Data Availability

The datasets generated and/or analyzed during the current study are not publicly available. The anonymized data can be obtained from two sources. Firstly, access to the anonymized data is given by the local government, which provided financial support for the current study. Secondly, the anonymized data are available from the corresponding author upon reasonable request and with permission of the Fangxing community and Qili Tang community and the Hefei municipality.
